# Novel technique in intraoperative localisation of skin cancer metastasis using ultrasound guidance: a case report

**DOI:** 10.1007/s12672-025-02463-w

**Published:** 2025-05-16

**Authors:** Nadin Hawwash, Amr Fadel, Niranjan Bista, Damian Mullan, Damir Kosutic

**Affiliations:** 1 School of Medical Sciences, Faculty of Biology, Medicine and Health, University of Manchester, Manchester, UK; 2 Cancer Research UK Manchester Cancer Research Centre, Manchester, UK; 3 Departments of Plastic Surgery, The Christie NHS Foundation, Manchester, UK; 4 Departments of Radiology, The Christie NHS Foundation, Manchester, UK

**Keywords:** Intraoperative ultrasound, Image-guided surgery, Case report, Resection

## Abstract

**Background:**

Localisation of metastatic squamous cell carcinoma (SCC) often poses intraoperative challenges. There is limited description of surgical practices to address these difficulties in the literature. Low-frequency ultrasound use intraoperatively may enhance tumour detection and facilitate complete resection.

**Case presentation:**

We present the case of a 78-year-old male with right-sided intra-parotid metastatic SCC requiring surgical excision. This was completed under intraoperative ultrasound scan guidance. Preoperative whole-body PET-CT and MRI of the head were inadequate for confirming accurate lesion localisation regarding the depth of invasion and facial nerve involvement. Intraoperative ultrasound performed by a consultant radiologist guided the metastasectomy by confirming lesion boundaries, navigating safe excision by sparing the facial nerve branches and facilitating the avoidance of more radical resection. Full resection with no residual disease was confirmed intraoperatively with the ultrasound.

**Conclusion:**

We propose using ultrasound guidance intraoperatively to aid localisation and excision of metastatic disease in anatomically challenging sites.

## Introduction

Complete and accurate resection of isolated metastatic and oligometastatic disease can be a surgical challenge in certain anatomical areas due to the proximity of important structures that are not easily identified with usual imaging modalities. In addition, when limited resections are attempted to reduce unnecessary morbidity, it can be challenging for the operating surgeon to ascertain whether complete clearance has been achieved under these circumstances. Where resections are found to be incomplete and margins to be positive on post-operative histology, subsequent surgery is required to ensure complete resection, increasing patient morbidity, costs and operating time. This is particularly true for intra-parotid lesions that grow towards the infratemporal fossa. Although total parotidectomy would be a desired surgery in these cases, often, this is not possible due to patients not being fit for a longer procedure or not accepting the risks of facial nerve palsy. We propose using intra-operative low-resolution ultrasound navigation to ensure objectively visualised complete removal of such lesions and reduce potential morbidity for the patient whilst reducing operating time (1).

## Materials and methods

**Fig. 1:**
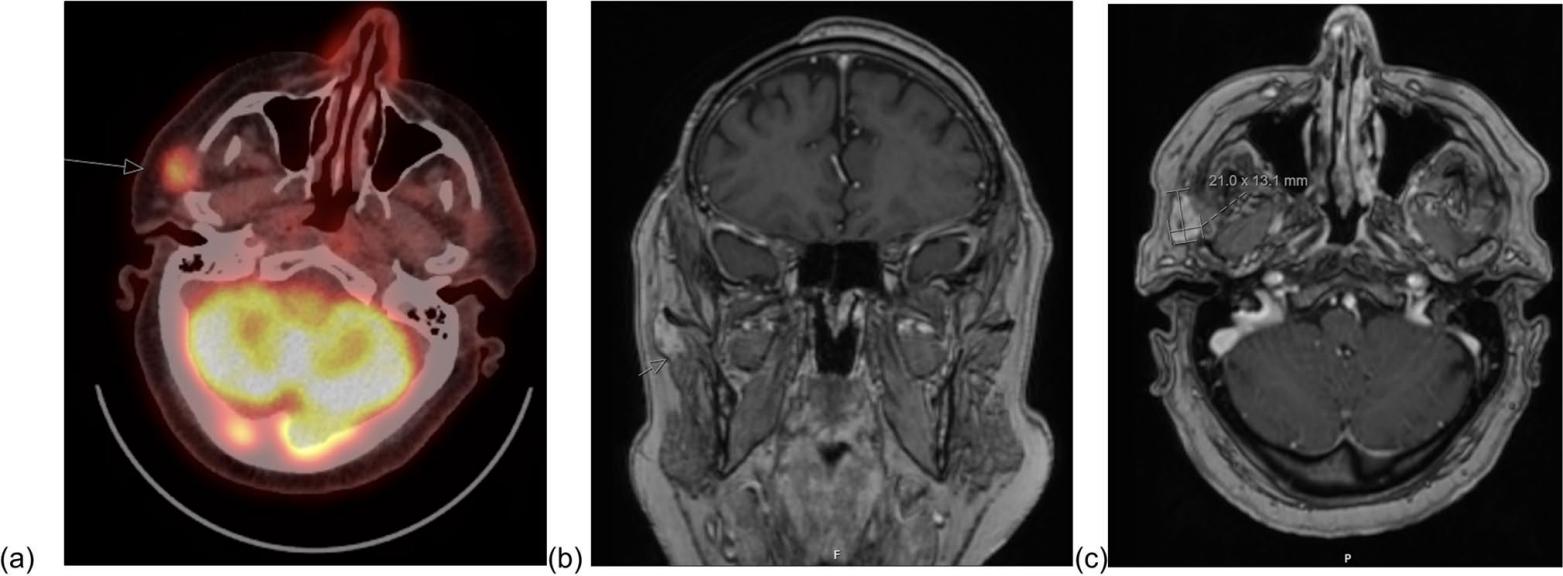
Pre-operative imaging **a** Fused Axial Positron Emission Tomography-Computed Tomography (PET-CT), **b** and **c** magnetic resonance imaging (MRI) of the head with contrast

**Fig. 2:**
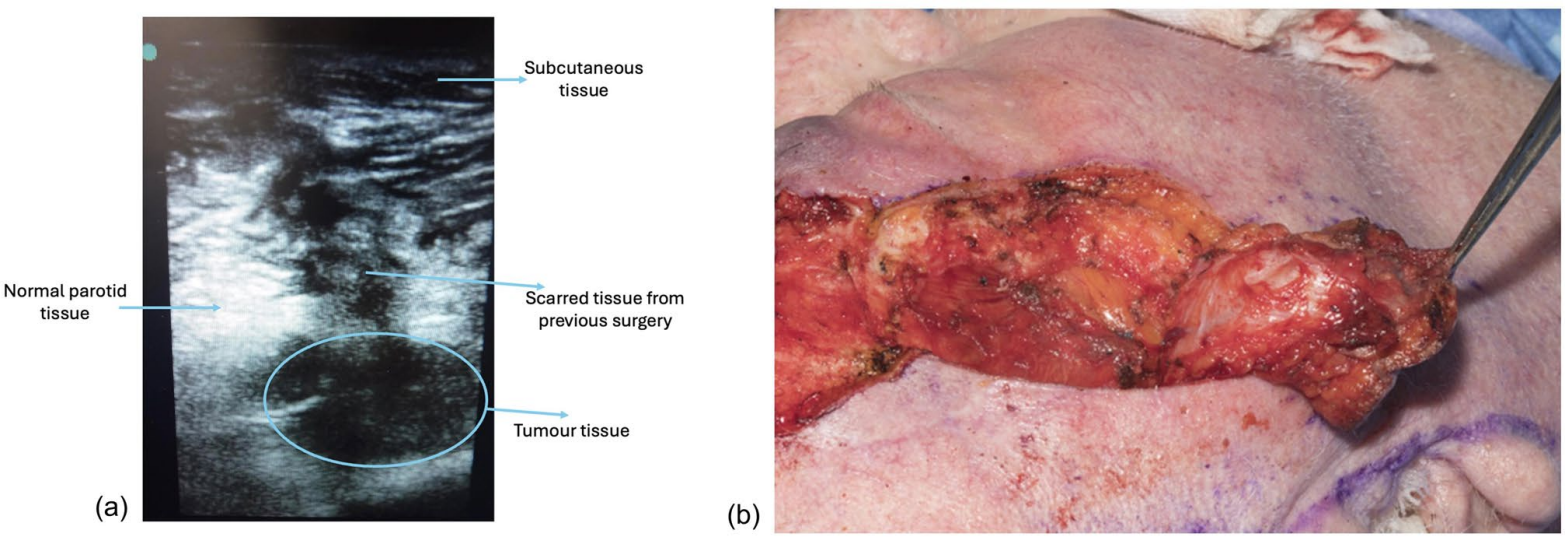
Intraoperative images including (a) ultrasound scan and (b) tumour resection and residual defect

We present a case of a 78-year-old man diagnosed with oligo-metastatic right-sided SCC with involvement of the right temple, right intra-parotid and right frontal regions with complex isolated multifocal sites of disease. The primary tumour diagnosed 8 months prior was a poorly differentiated biopsy-proven subcutaneous SCC in the right temple which was excised completely and the area was irradiated postoperatively. The patient had significant mortality risks as per the anaesthetic assessment; however, the patient still opted for surgery requiring oligo-metastasectomy. Preoperative imaging to localise the tumour included whole-body Positron Emission Tomography-Computed Tomography (PET-CT) (Fig. 1a). Magnetic resonance imaging (MRI) of the head with contrast was then used to locate the metastases further (Fig. 1b). This was followed by ultrasound-guided fine needle aspiration of the right parotid gland three weeks before surgery for cytological confirmation. An irregular 1.2 cm × 2.0 cm hypoechoic lesion in the right pre-auricular region was identified with ultrasound, and biopsy confirmed metastatic SCC alongside two other lesions as described above. Preoperative imaging was sufficient in localising the supraorbital and right temple SCC but insufficient in confirming accurate localisation of the nodal metastases in the parotid region. Therefore, intraoperative ultrasound visualisation (Fig. 2a) performed by a consultant radiologist was required to confirm the boundaries of the lesion and margins of excision, including the depth of invasion—which was not well visualised in the MRI scan and located in the right parotid gland lymph nodes—and to confirm that residual nodes did not contain any SCC metastases. Regarding the ultrasonographic technique, a 40-kHz ultrasound probe was used. Histopathology verified complete resection and negative surgical margins in pathology, confirming intraoperative US findings.

## Results

The ultrasound scan confirmed the full resection of metastatic disease intraoperatively. The intraoperative ultrasound scan navigated the safe excision of metastatic disease and with the use of intraoperative facial nerve monitoring helped preserve the facial nerve branches, reduce the extent of resection, and prevent facial nerve palsy. Immediately following resection, the ultrasound confirmed no residual tumour was left behind (Fig. 2b). The patient remained free of cancer for 6 months but subsequently developed intraorbital metastasis that would necessitate enucleation. The patient decided against further surgery, systemic treatment or radiotherapy.

## Discussion

Collaborative multidisciplinary intraoperative efforts involving radiology and plastic surgery provided essential real-time guidance through live visualisation of tumour boundaries and critical soft tissue structures, including nerves and vessels. While the duration of surgery was extended, it was more efficient, easily accessible, cost-effective, and associated with lower morbidity than a subsequent surgery for an incomplete resection. A previous prospective study of 60 patients with metastatic SCC found preoperative FDG PET to have a higher sensitivity and specificity compared with MRI and CT methods for detecting lymph node metastases of cancers in the head and neck (2). Chauhan et al. (2012) further found FDG-PET-CT to be more accurate with higher sensitivity (71.43%) and specificity (96.67%) compared with ultrasound (sensitivity 4.76% and specificity 93.33%) or contrast-enhanced CT staging (sensitivity 23.80% and specificity 93.33%) in N0 neck in head and neck squamous cell carcinoma (6). However, in our experience, despite robust pre-operative localisation with PET-CT and MRI, live intraoperative ultrasonography proved to be a useful tool for facilitating tumour detection, complete metastasectomy and confirming full resection of metastatic disease by identifying the absence of tissue heterogeneity in the tumour bed (typically indicative of residual lesion), while also reducing morbidity for the patient in anatomically difficult sites, such as the deep lobe of the parotid and infratemporal fossa. This approach has previously been effective in parotid tumour resection by Stetter et al. (2006) and the deep resection of an SCC of the tongue by de Koning et al. to get intraoperative feedback on deep resection margins and achieve desirable surgical margins (7, 8). Adriaasens et al. recently used this intraoperative ultrasound technique to measure tumour-free margins in SCC resections of the buccal mucosa, further demonstrating its feasibility in practice (9). Intraoperative emerging imaging techniques such as ex-vivo fluorescence and reflectance confocal microscopy have been used during Mohs micrographic surgery, but these techniques require specialised equipment and are limited to very superficial layers, unlike ultrasound imaging (5).

Ultrasound imaging has its limitations, particularly for deeper tumours or patients with higher BMI, as image resolution is lower with lower-frequency probes, and manoeuvrability may restrict the field of view (3). Intraoperative ultrasound heavily relies on the radiologist’s availability and expertise in accurately identifying structures, especially when there may be anatomical distortion during surgery, which may not be feasible at all sites. Garset-Zamani et al. (2024) demonstrated the practicality and benefits of surgeon-performed intraoperative transoral ultrasound imaging, but additional training and ultrasound probe availability may be limited (4). Overall, we believe intraoperative ultrasound-guided surgery could be applied to other anatomically challenging areas to ensure the adequacy of resection. This relatively quick, simple, non-invasive and effective technique could guide surgical procedures in selected patients with isolated or oligometastatic disease. However, accessibility across institutions may be limited given skill set and equipment availability requirements. A multicentre randomised trial is required to validate the added benefit of intraoperative USS imaging for complete resection of isolated metastatic and oligometastatic disease.

## Conclusions

We believe the use of intraoperative ultrasound visualisation to guide and confirm the excision of skin cancer metastasis in difficult locations can be a useful tool to avoid unnecessary damage to critical structures and overall patient morbidity.

## Data Availability

Data sharing does not apply to this article as no datasets were generated or analysed during the current study.
